# Supplementary material for: Observation of momentum-band topology in PT-symmetric Floquet lattices
Source: Nat Commun. 2025 Nov 13;16:9975. doi: 10.1038/s41467-025-64915-9 (PMC12615797; doi:10.1038/s41467-025-64915-9)
Supplement: Supplementary file 1 — Supplementary Information [file 41467_2025_64915_MOESM1_ESM.pdf]

Supplementary Information for

"Observation of momentum-band topology in PT-symmetric Floquet lattices"

Shuaishuai Tong[1], Qicheng Zhang[1], Gaohan Li[1], Kun Zhang[1], Chun Xie[1], and Chunyin Qiu[1,*]

[1] *Key Laboratory of Artificial Micro- and Nano-Structures of Ministry of Education and School of Physics and Technology, Wuhan University, Wuhan 430072, China*

[*]*To whom correspondence should be addressed: cyqiu@whu.edu.cn*

Contents

Supplementary Note 1. Enlarged Floquet Hamiltonian and effective Dirac model 3

Supplementary Note 2. Quantized EBZ Berry phase protected by PT-symmetry......................6

Supplementary Note 3. Momentum spectra under time-periodic boundary conditions 7

Supplementary Note 4. Additional evidence for Floquet momentum gap.................................9

Supplementary Note 5. Momentum sensitivity of the temporal TIM......................................10

Supplementary Note 6. Raw data without compensating background loss 11

Supplementary references...12

Supplementary Note 1. Enlarged Floquet Hamiltonian and effective Dirac model

a) Enlarged Floquet Hamiltonian

In this section, we derive the approximate Dirac model around the momentum gaps. We start from the two-band PT-symmetric Floquet lattice with nearest-neighbor hopping w. The two atoms in each unit cell have gain and loss $\pm[\gamma_\mathrm{s} + \gamma_\mathrm{d}(t)]$, where $\pm\gamma_\mathrm{s}$ denote time-independent static gain and loss, and $\pm\gamma_\mathrm{d}(t) = \pm\gamma \mathrm{S}(t)$ with $\mathrm{S}(t) = \mathrm{sgn}[\cos(2\pi\Omega t)]$ represent dynamic gain and loss. The momentum-space Hamiltonian of this periodically driven lattice reads

$$\mathbf{H}(t) = \begin{bmatrix} i\gamma_\mathrm{s} + i\gamma_\mathrm{d}(t) & w(1+e^{ik}) \\ w(1+e^{-ik}) & -i\gamma_\mathrm{s} - i\gamma_\mathrm{d}(t) \end{bmatrix}. \quad \text{(S1)}$$

Wave dynamics in this lattice is governed by the time-dependent Schrödinger equation

$$i\partial_t|\phi(t)\rangle = 2\pi\mathbf{H}(t)|\phi(t)\rangle. \quad \text{(S2)}$$

Since $\mathbf{H}(t+T) = \mathbf{H}(t)$, with $\mathrm{T} = 1/\Omega$, a solution to the time-dependent Schrödinger equation can be written in a Floquet form

$$|\phi(t)\rangle = e^{i2\pi Et}|u(t)\rangle, \quad \text{(S3)}$$

with $|u(t+T)\rangle = |u(t)\rangle$ and E being the quasi-energy, which can be calculated by seeking the solution of the following eigen-problem

$$2\pi E|u(t)\rangle = [2\pi\mathbf{H}(t) + i\partial_t]|u(t)\rangle\,. \quad \text{(S4)}$$

By expanding $|u(t)\rangle = [\psi_+(t), \psi_-(t)]^T$ and $\mathbf{H}(t)$ into time Fourier series

$$|u(t)\rangle = \left(\sum \psi_+^n e^{i2\pi n\Omega t}, \sum \psi_-^n e^{i2\pi n\Omega t}\right)^\mathrm{T}, \quad \text{(S5a)}$$

$$\mathbf{H}(t) = \sum_{m=-\infty}^{\infty} \mathbf{H}_m e^{i2\pi m\Omega t}\,, \quad \text{(S5b)}$$

where $\Omega = 1/T$ and $\psi_\pm^n$ ($\mathbf{H}_m$) is the nth (mth) order Fourier component of the wavefunction (Hamiltonian). By truncating $|u(t)\rangle$ to finite Floquet harmonics (from $\psi_\pm^{-N} e^{-i2\pi N\Omega t}$ to $\psi_\pm^N e^{i2\pi N\Omega t}$), we obtain

$$E|\psi\rangle = \mathcal{H}|\psi\rangle, \quad \text{(S6)}$$

where $|\psi\rangle = (\psi_+^N, \psi_-^N, \ldots, \psi_+^{+1}, \psi_-^{+1}, \psi_+^0, \psi_-^0, \psi_+^{-1}, \psi_-^{-1}, \ldots, \psi_+^{-N}, \psi_-^{-N})^\mathrm{T}$, and $\mathcal{H}$ is the time-independent enlarged Floquet Hamiltonian

$$\mathcal{H}=\begin{pmatrix} \mathbf{H_0}-N\Omega\mathbf{I} & \mathbf{H_1} & \mathbf{H_2} & \cdots & & \cdots & \mathbf{H}_{2N} \\ \mathbf{H}_{-1} & \ddots & \ddots & \ddots & \cdots & & \vdots \\ \mathbf{H}_{-2} & \ddots & \mathbf{H_0}-\Omega\mathbf{I} & \mathbf{H_1} & \mathbf{H_2} & \vdots & \\ \vdots & \ddots & \mathbf{H}_{-1} & \mathbf{H_0} & \mathbf{H_1} & \ddots & \vdots \\ & \vdots & \mathbf{H}_{-2} & \mathbf{H}_{-1} & \mathbf{H_0}+\Omega\mathbf{I} & \ddots & \mathbf{H} \\ \vdots & & \dots & \ddots & \ddots & \ddots & \mathbf{H_1} \\ \mathbf{H}_{-2N} & \cdots & & \cdots & \mathbf{H}_{-2} & \mathbf{H}_{-1} & \mathbf{H_0}+N\Omega\mathbf{I} \end{pmatrix}, \tag{S7}$$

The diagonal block matrix element $\mathbf{H}_0+n\Omega\mathbf{I}$ denotes the nth-order Floquet replica. Specifically, for the system characterized by Eq. (S1), we have $\mathbf{H}_0=\begin{bmatrix} i\gamma_{\mathrm{s}} & w+we^{ik} \\ w+we^{-ik} & -i\gamma_{\mathrm{s}} \end{bmatrix}$, and $\mathbf{H}_{n\,(n\neq 0)}=\mathrm{sinc}(n\pi/2)\begin{bmatrix} i\gamma & 0 \\ 0 & -i\gamma \end{bmatrix}$.

b) Effective Dirac model

Physically, Floquet momentum gaps originate from the interplay between different Floquet replicas. For simplicity, we consider a weak periodic drive with a small $|\gamma/w|$ and a large $|\Omega/w|$, where only nearest-neighbor Floquet replicas (e.g., $\mathbf{H}_0$ and $\mathbf{H}_0+\Omega\mathbf{I}$) interact with each other. In this case, near the quasi-energy $E=\Omega/2$ the system can be described by

$$\begin{bmatrix} \mathbf{H}_0+\frac{\Omega}{2}\mathbf{I} & \mathbf{H}_1 \\ \mathbf{H}_1 & \mathbf{H}_0-\frac{\Omega}{2}\mathbf{I} \end{bmatrix}\tilde{\psi}+\frac{\Omega}{2}\mathbf{I}\tilde{\psi}=E\tilde{\psi}. \tag{S8}$$

with $\tilde{\psi}=[\psi_+^{+1},\psi_-^{+1},\psi_+^0,\psi_-^0]$. For simplicity, we set $\gamma_{\mathrm{s}}=0$ as it hardly affects the Floquet momentum gap. Providing that $(E-\Omega/2)\to 0$ around the Floquet momentum gap (due to the non-Hermitian chiral symmetry $\sigma_z H_{\mathrm{eff}}^{\dagger}\sigma_z=-H_{\mathrm{eff}}$), the 4×4 Hamiltonian in Eq. (S8) can be simplified to an effective 2×2 Hamiltonian by eliminating ψ_+^{+1} and ψ_-^0 (Ref. S1). This gives rise to

$$\frac{1}{\Omega}\begin{bmatrix} -2w^2-2w^2\cos(k)+\frac{\Omega^2}{4}+\left(\frac{2\gamma}{\pi}\right)^2 & -i4rw(1+e^{-ik})/\pi \\ -i4rw(1+e^{ik})/\pi & 2w^2+2w^2\cos(k)-\frac{\Omega^2}{4}-\left(\frac{2\gamma}{\pi}\right)^2 \end{bmatrix}\psi'=\delta E\psi', \tag{S9}$$

where $\delta E=E-\Omega/2$ and $\tilde{\psi}'=[\psi_-^{+1},\psi_+^0]$. When $\gamma=0$, there is a Floquet Dirac degenerate

point with $\delta E = 0$ at a momentum $k_\mathrm{D} = 2\cos^{-1}(\frac{\Omega}{4w})$. Therefore, by setting $\delta k = k - k_\mathrm{D}$ and applying a linear approximation around the Dirac point, we have

$$\frac{1}{\Omega}\begin{bmatrix} -2w^2\sin(k_\mathrm{D})\delta k & -i4\gamma w(1+e^{-ik_\mathrm{D}})/\pi \\ -i4\gamma w(1+e^{ik_\mathrm{D}})/\pi & 2w^2\sin(k_\mathrm{D})\delta k \end{bmatrix}\tilde{\psi}' = \delta E\tilde{\psi}'. \tag{S10}$$

Equation (S10) can be represented utilizing Pauli matrix

$$(-im\sigma_x - v_\mathrm{D}\delta k\sigma_z)\psi_\mathrm{D} = \delta E\psi_\mathrm{D}, \tag{S11}$$

where the imaginary Dirac mass $m = 8\gamma w(\pi\Omega)^{-1}\cos\left(\frac{k_\mathrm{D}}{2}\right) = 2\gamma\pi^{-1}$, the Dirac velocity $v_\mathrm{D} = 2w^2\Omega^{-1}\sin(k_\mathrm{D}) = \sqrt{w^2 - (\Omega/4)^2}$, and $\psi_\mathrm{D} = \mathbf{M}\psi'$ with the transformation matrix $\mathbf{M} = \begin{bmatrix} e^{ik_\mathrm{D}/2} & 0 \\ 0 & 1 \end{bmatrix}$. As shown in Fig. S1, the Dirac model described by Eq. (S11) exhibits excellent agreement with our lattice model near the Floquet momentum gap.

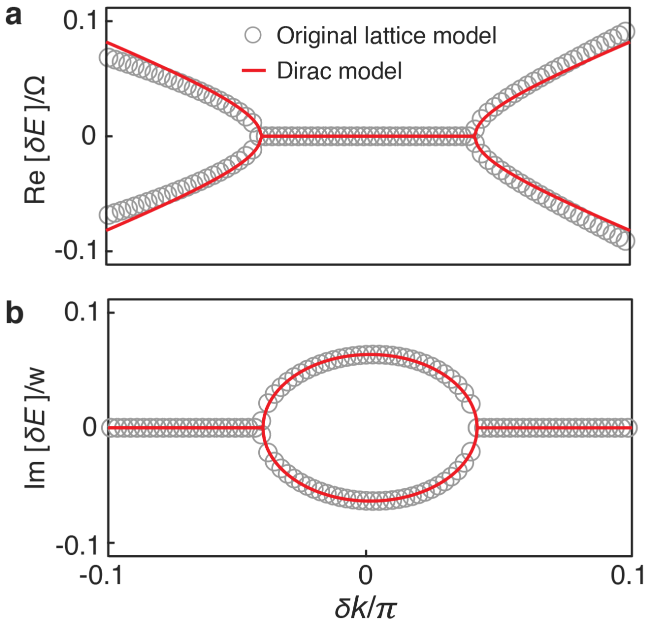

Supplementary Fig. 1. Band structure of the Dirac model. Real (**a**) and imaginary (**b**) energy-momentum spectra of the original lattice model and Dirac model near the Floquet momentum gap, calculated with parameters $\Omega = 3.5w$, $\gamma_s = 0$, and $\gamma = 0.1w$.

It is of particular interest that multiplying the Eq. (S11) with σ_z yields

$$v_\mathrm{D}\delta k\psi_\mathrm{D} = \left(m\sigma_y - \delta E\sigma_z\right)\psi_\mathrm{D} \tag{S12}$$

It resembles the form of a Hermitian Dirac Hamiltonian but with energy and momentum swapped. In particular, the nonzero imaginary Dirac mass m opens a momentum gap with topology dependent on the sign of m.

Now we consider a temporal domain-wall structure where the imaginary Dirac mass $m(t)$ reverses the sign at $t = 0$

$$m(t) = \begin{cases} -m_0, & t < 0 \\ m_0, & t > 0 \end{cases}, \tag{S13}$$

with $m_0 > 0$. For $\delta k = 0$, solving the Schrödinger equation $i\frac{\partial}{\partial t}|\psi_{\mathrm{D}}(t)\rangle = i2\pi m(t)\sigma_x|\psi_{\mathrm{D}}(t)\rangle$ gives a temporal localized state

$$|\boldsymbol{\psi}(t)\rangle \propto e^{-2\pi m_0|t|}[1,-1]^{\mathrm{T}}, \tag{S14a}$$

and an anti-localized state

$$|\boldsymbol{\psi}(t)\rangle \propto e^{2\pi m_0|t|}[1,1]^{\mathrm{T}}. \tag{S14b}$$

The localized state, exhibiting a temporal localization at $t = 0$, aligns well with the topological interface mode in our lattice model (Fig. 2f, main text), whereas the anti-localized state diverges as $|t| \to \infty$ and is physically inaccessible.

Supplementary Note 2. Quantized EBZ Berry phase protected by PT-symmetry

The effective Hamiltonian $\mathbf{H}_{\mathrm{eff}}$ of our lattice exhibits a PT-symmetric momentum band between the Floquet momentum gap and the static momentum gap. In this section, we show the connection between the states' PT eigenvalue $e^{i\varphi(E)}$ and the Berry phase in energy Brillouin zone (EBZ) θ_E. For the PT-symmetric momentum band with eigenstates $\psi(E)$, the PT eigenvalue is given by $e^{i\varphi(E)}\psi(E) = \mathbf{PT}\psi(E)$ (Ref. S2). Since $\mathbf{PT}$ is antiunitary, we have

$$\partial_E(\psi^\dagger\psi) = 0 \Rightarrow \psi^\dagger\partial_E\psi = -(\partial_E\psi^\dagger)\psi = -(\partial_E\psi^\dagger)(\mathbf{PT})^\dagger\mathbf{PT}\psi = -[\partial_E(\mathbf{PT}\psi)^\dagger]\mathbf{PT}\psi \Rightarrow$$

$$\psi^\dagger\partial_E\psi = -(e^{i\varphi}\psi)^\dagger\partial_E(e^{i\varphi}\psi) = -i\partial_E\varphi - \psi^\dagger\partial_E\psi \Rightarrow i\psi^\dagger\partial_E\psi = \frac{1}{2}\partial_E\varphi. \tag{S15}$$

Therefore, the energy-space Berry connection $A_E = i\psi^\dagger\partial_E\psi$ of a given PT-symmetric band equals to half the gradient of the states' phase factor $\varphi(E)$ under $\mathbf{PT}$ transformation. This further gives the Berry phase

$$\theta_E = \oint_{\mathrm{EBZ}} A_E dE = \frac{1}{2}\oint_{\mathrm{EBZ}} \partial_E\varphi dE, \tag{S16}$$

Ultimately, the periodicity of the Floquet eigenfunction in the EBZ, $\varphi(E+\Omega) = \varphi(E)$, enforces a quantized and gauge-invariant Berry phase $\theta_E = 0$ or π. Note that $\varphi(E)$ is gauge-dependent. For simplicity, we choose a gauge such that the eigenfunction at site 1 is real-valued in Fig. 4c of the main text.

Supplementary Note 3. Momentum spectra under time-periodic boundary conditions

To demonstrate the temporally localized state between two lattices with distinct momentum-gap topologies, we construct a temporal domain-wall structure shown in Fig. S2, where $\gamma = \gamma_{\mathrm{L}}$ for $-NT < t < 0$ and $\gamma = \gamma_{\mathrm{R}}$ for $0 < t < NT$, with N characterizing temporal duration of the time-domain wall structure. The time-evolution operator of this temporal domain-wall structure reads $\mathbf{U}_{\mathrm{DW}}(k) = \mathbf{U}_{\mathrm{R}}^{N}(k)\mathbf{U}_{\mathrm{L}}^{N}(k)$, with $\mathbf{U}_{\mathrm{L,R}}(k) = e^{-i2\pi\mathcal{T}\int_0^T \mathbf{H}_{\mathrm{L,R}}(k,t)dt}$. To solve for the momentum spectrum and eigenstates of the temporal domain-wall structure, we apply the temporal periodic boundary condition $\psi_{t=NT} = \psi_{t=-NT}$, and the spatial Bloch boundary condition $\psi_{x=m+1} = e^{ik}\psi_{x=m}$, where m indices the spatial position of unit cell. Given the connection between $\psi_{t=NT}$ and $\psi_{t=-NT}$ through the time evolution operator $\mathbf{U}_{\mathrm{DW}}(k)$ of the entire temporal domain-wall structure, i.e., $\psi_{t=NT} = \mathbf{U}_{\mathrm{DW}}(k)\psi_{t=-NT}$, the temporal periodic boundary condition $\psi_{t=NT} = \psi_{t=-NT}$ enforces that the eigenvalue of $\mathbf{U}_{\mathrm{DW}}(k)$ must be 1. According to the definition of quasi-energy, $e^{-i2NT\varepsilon}\psi = \mathbf{U}_{\mathrm{DW}}\psi$, we have the quasi-energies $\varepsilon = 0$ for all eigenstates of the domain-wall structure. Solving the eigen-problem $\mathbf{U}_{\mathrm{DW}}(k)\psi = u(k)\psi$, we obtain all the momenta that satisfy $u(k_{\mathrm{P}}) = 1$. The corresponding time-domain eigenstates can be calculated utilizing the eigenstate ψ by $\psi(t) = e^{-i2\pi\mathcal{T}\int_{-NT}^{t} \mathbf{H}_{\mathrm{L}}(k_{\mathrm{P}},t)dt}\psi$ for $-NT < t < 0$ and $\psi(t) = e^{-i2\pi\mathcal{T}\int_0^t \mathbf{H}_{\mathrm{R}}(k_{\mathrm{P}},t)dt}\mathbf{U}_{\mathrm{L}}^{N}(k_{\mathrm{P}})\psi$ for $0 < t < NT$.

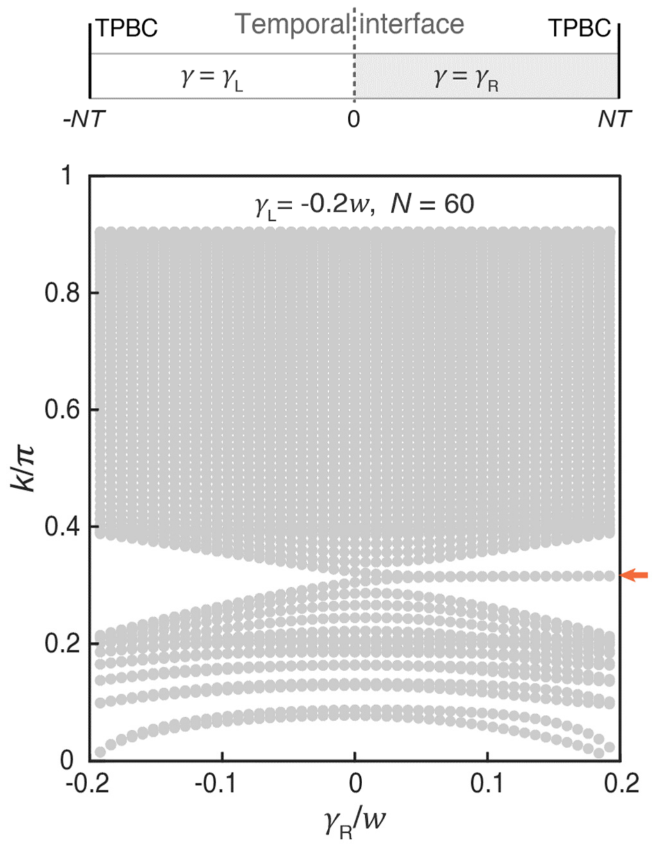

Supplementary Fig. 2. Momentum spectrum of a temporal domain-wall structure under time-periodic boundary condition (TPBC), calculated with fixed $N = 30$ and $\gamma_L = -0.2w$.

Figure S2 presents the momentum spectrum of the temporal domain-wall structure with fixed $\gamma_L = -0.2w$ but varied γ_R. For $\gamma_R < 0$, lattices on both sides of the time interface are topologically trivial, and thus no isolated states emerge within the momentum gap. On the contrary, for $\gamma_R > 0$, the lattice on the right side becomes topologically nontrivial, leading to the appearance of a pair of isolated states within the momentum bandgap (indicated by the yellow arrow). These results are consistent with the phase diagram shown in Fig. 2d of the main text.

Supplementary Note 4. Additional evidence for Floquet momentum gap

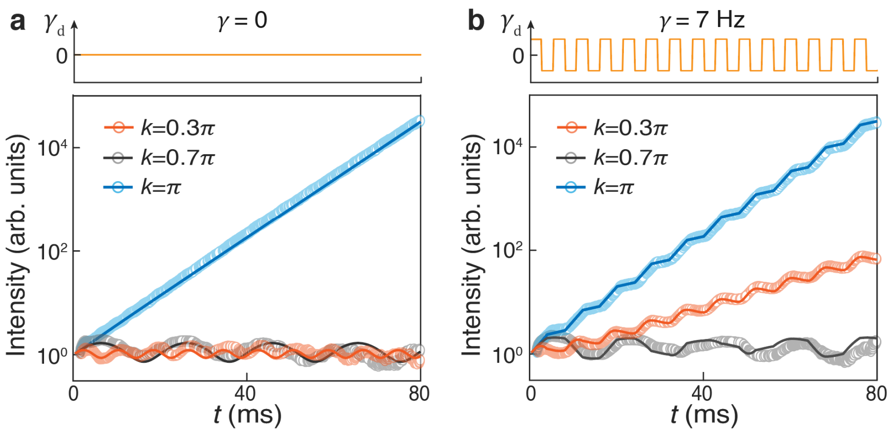

Supplementary Fig. 3. Time-evolution of the sound intensities $|\psi_1(t)|^2$ measured for three typical momenta. a and **b** present comparative results for the systems with $\gamma = 0$ and $\gamma = 7$ Hz, respectively. The experimental data (circles), matching well with the simulations (solid lines), showcase additional evidence for the Floquet momentum gap induced by the temporal modulation of gain and loss.

To intuitively confirm the existence of the Floquet momentum gap, we measured the time evolutions of sound intensities $|\psi_1(t)|^2$ at three typical momenta: $k = 0.3\pi$, 0.7π, and π. Figures S3a,b present comparative results for the systems with $\gamma = 0$ and $\gamma = 7$ Hz, respectively. The experimental data, aligning with the simulation results, show that both systems exhibit similar extended bulk states at $k = 0.7\pi$ and exponentially growing static momentum-gap states at $k = \pi$. However, at $k = 0.3\pi$, exponential growth of states occurs in the $\gamma = 7$ Hz system but not in the $\gamma = 0$ system, providing clear evidence for the existence of the Floquet momentum gap.

Supplementary Note 5. Momentum sensitivity of the temporal TIM

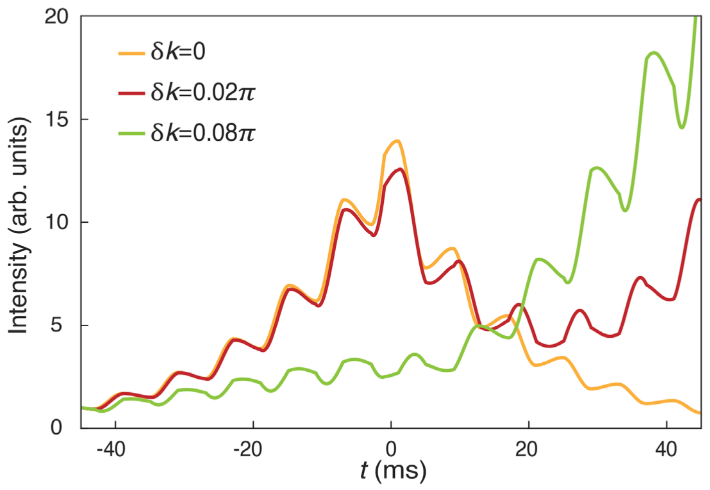

Supplementary Fig. 4. Sound intensity evolutions simulated for the time-domain wall systems at momenta $k = k_D + \delta k$, with $\delta k = 0$, $\delta k = 0.02\pi$, and $\delta k = 0.08\pi$. The results illustrate a high momentum sensitivity of the acoustic time-localized interface state.

The temporal TIM is isolated within the Floquet momentum gap, similar to how the spatial TIM is confined within the energy bandgap. However, unlike the energy gap, which only supports evanescent waves that decay in space, the momentum gap supports growing modes whose amplitudes increase exponentially over time (as observed in Fig. S3). This behavior can disrupt the localization around the temporal interface. To illustrate this, Figure S4 presents the sound intensity evolutions for cases around the momentum k_D. It shows that the perfect interface state at k_D, which exponentially grows and then decays after $t = 0$, is clearly disrupted by a slight momentum deviation $\delta k = 0.02\pi$, with the amplitude growing undesirably for $t > 20$ ms. As δk increases to 0.08π, the interface state is completely destroyed and behaves as a monotonically growing state over time. This demonstrates that the time-localized interface state is highly sensitive to the momentum selection. This momentum sensitivity, essentially arising from the coexistence of growing in-gap modes, contributes to the visible deviation between the experimental and simulated results observed in Fig. 5b of the main text. Interestingly, as a bonus, such sensitivity to system parameters may offer a new approach for sensor design.

Supplementary Note 6. Raw data without compensating background loss

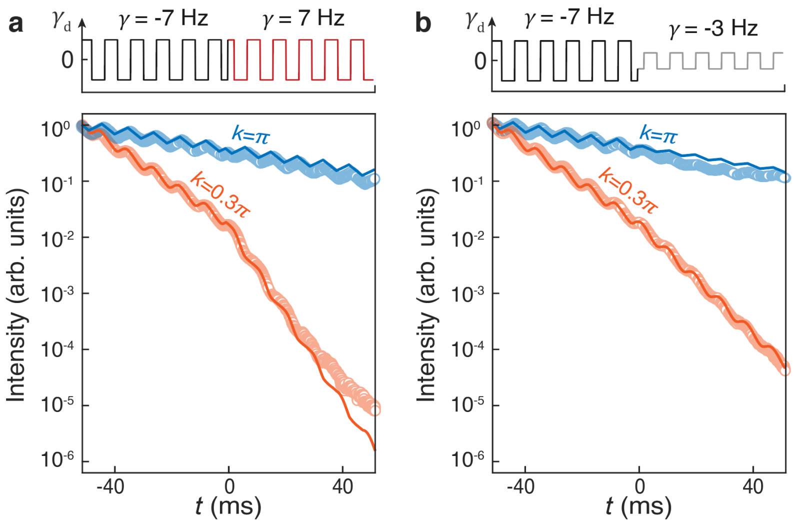

Supplementary Fig. 5. Raw data for the sound intensities presented in Fig. 5. a, Raw sound intensities for the nontrivial temporal interface at the momenta $k = 0.3\pi$ and $k = \pi$, with experimental data (circles) closely matching the simulation results calculated under a uniform background loss $\gamma_0 = -13$ Hz (lines). A base-10 logarithmic scale is used to better visualize the exponential behavior. **b,** Similar to **a**, but for the trivial temporal interface system.

The time-domain wavefunction under a uniform background loss γ_0 reads $\psi'(t) = e^{-i2\pi\mathcal{T}\int_0^t[\mathbf{H}(t)+i\gamma_0\mathbf{I}]dt}\psi_0 = e^{2\pi\gamma_0 t}e^{-i2\pi\mathcal{T}\int_0^t\mathbf{H}(t)dt}\psi_0 = e^{2\pi\gamma_0 t}\psi(t)$, i.e., the presence of γ_0 does not affect the essential physics of $\mathbf{H}(t)$ but introduces an extra exponential decay $e^{2\pi\gamma_0 t}$ in the lossless wavefunction $\psi(t)$. Mathematically, this decay can be compensated by multiplying an exponential factor $e^{-2\pi\gamma_0 t}$ to the wavefunction $\psi'(t)$. Figure S5 presents the raw sound intensities corresponding to the compensated data in Figs. 5b,d of the main text, compared with the simulated results. It shows that without compensating for the background loss, only a small kink is observed in the topological interface states.

Supplementary references

S1. Gail1, R. D., Goerbig, M. O., Guinea, F., Montambaux, G. & Castro Neto, A. H. Topologically protected zero modes in twisted bilayer graphene. Phys. Rev. B **84**, 045436 (2011).

S2. Stegmaier, A. et al. Topological Defect Engineering and Symmetry in Non-Hermitian Electrical Circuits. Phys. Rev. Lett. **126**, 215302 (2021).
